# Decolonising multimorbidity? research gaps in low and middle-income countries

**DOI:** 10.11604/pamj.2022.41.140.32104

**Published:** 2022-02-17

**Authors:** Rifqah Abeeda Roomaney, Brian Van Wyk, Victoria Pillay-Van Wyk

**Affiliations:** 1 Burden of Disease Research Unit, South African Medical Research Council, Cape Town, South Africa,; 2 School of Public Health, University of the Western Cape, Cape Town, South Africa

**Keywords:** Multimorbidity, low and middle-income countries, comorbidity

## Abstract

Multimorbidity is defined as the co-existence of multiple health conditions in one person. However, its use in research has been predominantly applied to non-communicable diseases, because research was conducted almost exclusively in developed countries. More recently, infectious diseases of long duration, such as human immunodeficiency virus (HIV), have also been included in the conceptualization of multimorbidity. While multimorbidity is a growing area of research globally; much less is known about the phenomenon in low and middle-income countries (LMICs) where disease burdens are heavily impacted by HIV. Health systems and services tend to be constrained in LMICs and information on disease patterns are important to better prioritize services. This commentary aims to describe the changing conceptualization of multimorbidity, the dearth of research into multimorbidity in LMICs and how the knowledge generated by research in LMICs can contribute to the global understanding of multimorbidity. LMICs can play a key role in the implementation of integration research.

## Commentary

**Introduction:** multimorbidity refers to the co-existence of multiple health conditions in an individual. Living with more than one disease condition impacts how affected individuals receive and manage their medical treatment, and also how health services are structured [1]. At the individual level, multimorbidity is associated with reduced wellbeing, a decreased quality of life and higher mortality rates. People with multimorbidity face a great “treatment burden” as their time and energy are impacted through accessing care from multiple providers. Those with multimorbidity have to self-manage complex treatment plans with multiple drugs, are less likely to adhere to treatment due to being overwhelmed, and some suffer from adverse drug reactions due to polypharmacy. Multimorbidity can be challenging for healthcare professionals to treat, due to the complexity of following numerous guidelines and challenges in delivering patient-centred care. In addition, patients with multimorbidity are usually excluded from drug trials, which can create uncertainty in treatment guidelines. From a health systems perspective, multimorbidity leads to increased healthcare utilization and healthcare costs. Most healthcare systems, clinical teams and clinical guidelines are organised around single diseases or single organ lines, further hindering patients´ access to integrated care. In this commentary, we argue that there is a dearth of research into multimorbidity in low and middle-income countries (LMICs) and that the knowledge generated from LMICs can contribute to a more nuanced and globally relevant understanding of multimorbidity.

**Defining the problem:** what “health conditions” are included in the conceptualization of multimorbidity has been subject to debate from as early as the year 2001. The definition of multimorbidity has been notoriously heterogeneous, and numerous institutions have attempted to define it. In 2013, the European General Practice Research Network presented a holistic definition whereby they described multimorbidity as the “combination of chronic disease with at least one other disease (acute or chronic) or biopsychosocial factor (associated or not) or somatic risk factor” [2]. In 2016, the World Health Organization (WHO) defined it as the “coexistence of two or more chronic conditions in the same individual” and further elaborated that they are referring to long-term health conditions which require complex and ongoing care [3]. Following a workshop held in South Africa in 2016, the Academy of Medical Sciences and the Academy of Science South Africa proposed a definition in line with the WHO whereby it was defined as the “co-existence of two or more chronic conditions” and added that these chronic conditions can include physical non-communicable disease of long duration, a mental health condition of long duration or an infectious disease of long duration such as HIV and TB [1]. Their definition clearly includes HIV, which is important because there is ambiguity in what is considered a “chronic disease” and whether it includes infectious diseases of long duration such as HIV or Hepatitis C. The workshop also noted that due to the high burden of disease due to HIV, multimorbidity may affect young people in low and middle-income countries.

**Why the focus on multimorbidity?:** while multimorbidity in its current conceptualization may be a relatively new concept, there have always been people that have suffered from more than one disease. The term multimorbidity was first used in Germany in 1976 and became internationally recognised in the 1990s [4]. The area of multimorbidity has seen an exponential rise in the number of articles published in the past two decades [4]. So, why has multimorbidity become such an important area of research? The attention to this area could be for various reasons. All over the world, but especially in high income countries, the general population is ageing. Many studies have shown that multimorbidity is associated with ageing; the older an individual gets, the higher chances of developing multiple diseases. This, in part, explains the focus on multimorbidity as it is often put in the same category as frailty, geriatrics and of managing diseases in the elderly. Another reason is access to high quality information. Researchers have been able to analyse large administrative datasets or electronic health records to better understand the burden of multimorbidity in their country. These datasets make it easier to track people that have multiple disease conditions or are taking medication for more than one disease.

**LMICs have been left behind:** the estimated prevalence of multimorbidity in LMICs is not much lower than that of high-income countries (30% versus 38%, respectively) [5]. Despite this, there is a dearth of studies on multimorbidity in LMICs. Only 5% of studies on multimorbidity were focused on or were set in LMICs [4]. A recent scoping review of multimorbidity studies in LMICs further revealed that the majority of multimorbidity studies were confined to only six middle-income countries (e.g. Brazil, China, South Africa, India, Mexico and Iran) [6]. This indicates that there is much to uncover regarding multimorbidity in LMICs, especially in low income countries.

Populations in LMICs are also ageing, the burden of non-communicable diseases is increasing rapidly, and this is coupled with existing burdens of infectious diseases. Infectious disease conditions form a substantial proportion of the burden of disease in LMICs compared to disease burdens in high income countries. The intersecting burden between non-communicable and infectious diseases has been highlighted in parts of the world, such as South Africa; where HIV prevalence is high and no longer a death sentence due to the availability of antiretroviral medication. While there has been success in integrating HIV and TB services and surveillance programmes, more work needs to be done to link these to other diseases; especially since many people living with HIV also suffer from other chronic diseases such as hypertension and common mental disorders. In addition, research has emerged to show that multimorbidity does not only affect the elderly. Due to the high burden of HIV in LMICs, researchers believe that younger people could develop multiple disease conditions because HIV tends to impact younger adults [1]. Some research indicates that people from lower socioeconomic settings may be more vulnerable to multimorbidity, which may also have implications for LMICs.

Health systems in LMICs are arguably more constrained and tend to have less robust routine health information systems, making it difficult to even identify whether multimorbidity is an issue. Also, in many LMICs, vertical programme structures often exist due to international donor requirements perpetuated by unequal power relations [7]. International donors have the power to influence monitoring and evaluation processes [7] which could further hinder the identification of multimorbidity as problematic in LMICs dependent on donor funding, if it does not fit the agenda of the donor. For example, in South Africa, it is difficult to monitor noncommunicable diseases (NCDs) as these diseases received little attention in the past [8]. Systems to monitor HIV and TB are comparatively better developed, in part, due to funding by international donors. While HIV and TB systems have been implemented in most public health facilities [8], data quality issues persist.

**Further research and the potential for innovation:** more research is needed into multimorbidity in LMICs as patterns of disease burdens may significantly differ from those in high income countries and could generate hypotheses on lesser known disease combinations. In doing so, we may need to tweak our existing conceptualization of multimorbidity (for example, the consideration of acute disease) so that the concept of multimorbidity is more meaningful to contexts outside of high income countries. There have been arguments against the inclusion of acute disease conditions for practical reasons or that its inclusion may inflate the prevalence of multimorbidity. Another reason could be that acute disease conditions may also not be of interest to where the majority of multimorbidity studies emanates from.

The definition of multimorbidity is loose and broad enough to include diseases such as HIV, TB and Hepatitis B, etc. However, more guidance is needed around when is the duration of a disease considered “long enough” or disease conditions of an episodic nature. Also, by completely excluding acute disease conditions from the concept of multimorbidity, we may end up limiting the research on emerging acute infectious diseases, like COVID-19. While mental health services have generally been overlooked in LMICs; in 2020, the WHO highlighted the need for increased effort to tackle the mental health burden of people with neglected tropical diseases [9]. Thus highlighting the intersection of two incredibly neglected areas of research and health service. Another area of concern to LMICs is injuries and disability due to interpersonal violence and road traffic injuries. We conducted a systematic review to identify studies that quantify the prevalence of multimorbidity in South Africa, and identify common disease clusters and identify research gaps [10].

LMICs need to be a part of the efforts to identify and manage multimorbidities. Countries such as South Africa are recognised as being on the cutting edge of implementation research regarding the integration of care. The South African government has planned to integrate care by capitalizing on existing HIV infrastructure, in an effort towards health systems strengthening. This would help to monitor, manage and integrate services for other important HIV co-morbidities such as diabetes, hypertension and mental disorders. Other examples of interventions in South Africa include the integrated chronic disease management model, the collaborative care model for common mental disorders comorbid with chronic conditions and the Practical Approach to Care Kit to support clinical decision-making and also integrates the routine care of common comorbidities.

**Funding information:** the work reported herein was made possible through funding by the Burden of Disease Research Unit at the South African Medical Research Council. R.A.R. conducted this research under the South African Medical Research Council through its Division of Research Capacity Development under the Internship Scholarship Programme from funding received from the South African National Treasury. The content hereof is the sole responsibility of the authors and does not necessarily represent the official views of the South African Medical Research Council or the funders. Grant number: NA.

## Conclusion

Studies on multimorbidity are very limited in LMICs - they are limited both in the number of studies that have been conducted but also in the depth of information available due to health information systems which limit data interoperability. These countries tend to lack the resources to show that multimorbidity is a problem (e.g. access to electronic health records) and when they do, they need to ensure that the disease conditions assessed are relevant to their respective settings. More studies are needed on multimorbidity in LMICs. The findings of these studies could be used to organise health systems more effectively and improve the healthcare experiences for those people living with multimorbidity in LMICs. By identifying disease burdens relevant to LMICs, interventions in one setting could be trialled in others. It could also be used to generate hypotheses and inform interventions to screen, detect and treat people; especially considering the emerging diabetes epidemic in LMICs. These findings may also be relevant to high income countries, where patterns of multimorbidity in LMICs could be similar to those in immigrant populations.
